# Elbow anatomy in perspective of joint capsule and surrounding aponeuroses: a narrative review

**DOI:** 10.1016/j.jseint.2024.01.006

**Published:** 2024-02-02

**Authors:** Akimoto Nimura, Haruhiko Shimura, Shota Hoshika, Atsuhiro Fukai, Keiichi Akita

**Affiliations:** a Department of Functional Joint Anatomy, Graduate School of Medical and Dental Sciences, Tokyo Medical and Dental University, Tokyo, Japan; b Department of Clinical Anatomy, Graduate School of Medical and Dental Science, Tokyo Medical and Dental University, Tokyo, Japan

**Keywords:** Anatomy, Elbow, Joint capsule, Coronoid process, Lateral collateral ligament, Lateral ulnar collateral ligament, Ulnar collateral ligament

## Abstract

**Background:**

Because of the proximity of several ligaments, aponeuroses, and capsule in the limited area of the elbow joint, the precise anatomy is difficult to understand. In the current narrative review, we focused on two anatomical perspectives: the capsular attachment and structures consisting of ligaments.

**Methods:**

Based on the previously performed studies regarding the elbow anatomy, a narrative review was prepared in terms of the capsular attachment and structures consisting of ligaments.

**Results:**

At the tip of the coronoid process, the joint capsule attaches roughly 6 mm distal to its tip with 6-12 mm length. On the lateral epicondyle of the humerus, the capsular attachment at the anterior part of the extensor carpi radialis brevis origin is narrower than the one distal to it. A single interpretation of the lateral collateral ligament is the capsulo-aponeurotic membrane, which is composed of the joint capsule intermingling with the supinator aponeurosis. The anterior bundle of the ulnar collateral ligament could be interpreted as the grossly separated collagenous structure from the tendinous complex, which is composed of the tendinous septum between the flexor digitorum superficialis and pronator teres muscle, the medial part of the brachialis muscle, and deep aponeurosis of the flexor digitorum superficialis muscle.

**Discussion:**

Based on these perspectives, ligaments could function as a “static-dynamic” stabilizer rather than a simple static one.

To discuss clinical issues in the elbow joint, such as instability of the lateral and medial sides and lateral and medial epicondylitis, anatomical knowledge in detail should be important. However, the proximity of several ligaments, aponeuroses, and joint capsules in the narrow region of elbow makes anatomical understanding difficult.

We previously analyzed the elbow anatomy based on the relationship between the intermuscular tendons, surrounding aponeuroses, and joint capsule.^7^^,^^10^^,^^18^^,^^27^ We particularly focused on two specific perspectives: attachment of joint capsules and structures consisting of ligaments, as described below. In the current review, we present our research topics on clinical anatomy in the elbow joint and discuss insights into pathogenesis and stabilizing mechanisms.

## The capsule of elbow joint and its attachment widths

Fractures of the ulnar coronoid process have been controversially discussed to fix or not using the Regan and Morrey classification.^25^ O’Driscoll et al^22^ suggested a novel classification of fracture variations according to the image obtained by computed tomography. Particularly, they divided the tip fracture (type 1) into two subtypes: 1) involving less than or equal to 2 mm of the coronoid and 2) involving larger than 2 mm. They proposed subtype 1 of tip fracture might not require the internal fixation. However, the anatomical evidence of suggestion was unclear, because the anatomical knowledge regarding the anterior capsule of elbow joint and its attachment were not understood.

In general, the joint capsule has not been thought to have an attachment width, ie, the capsule was assumed to be linearly attached to bones, because of its consistently thin and tiny structure. However, recent anatomical studies from perspective of joint capsule attachments revealed that the joint capsule attached with width and varied with locations in several joints such as the shoulder,^19^ knee,^17^ hand,^26^ and ankle.^1^

**Figure 1:**
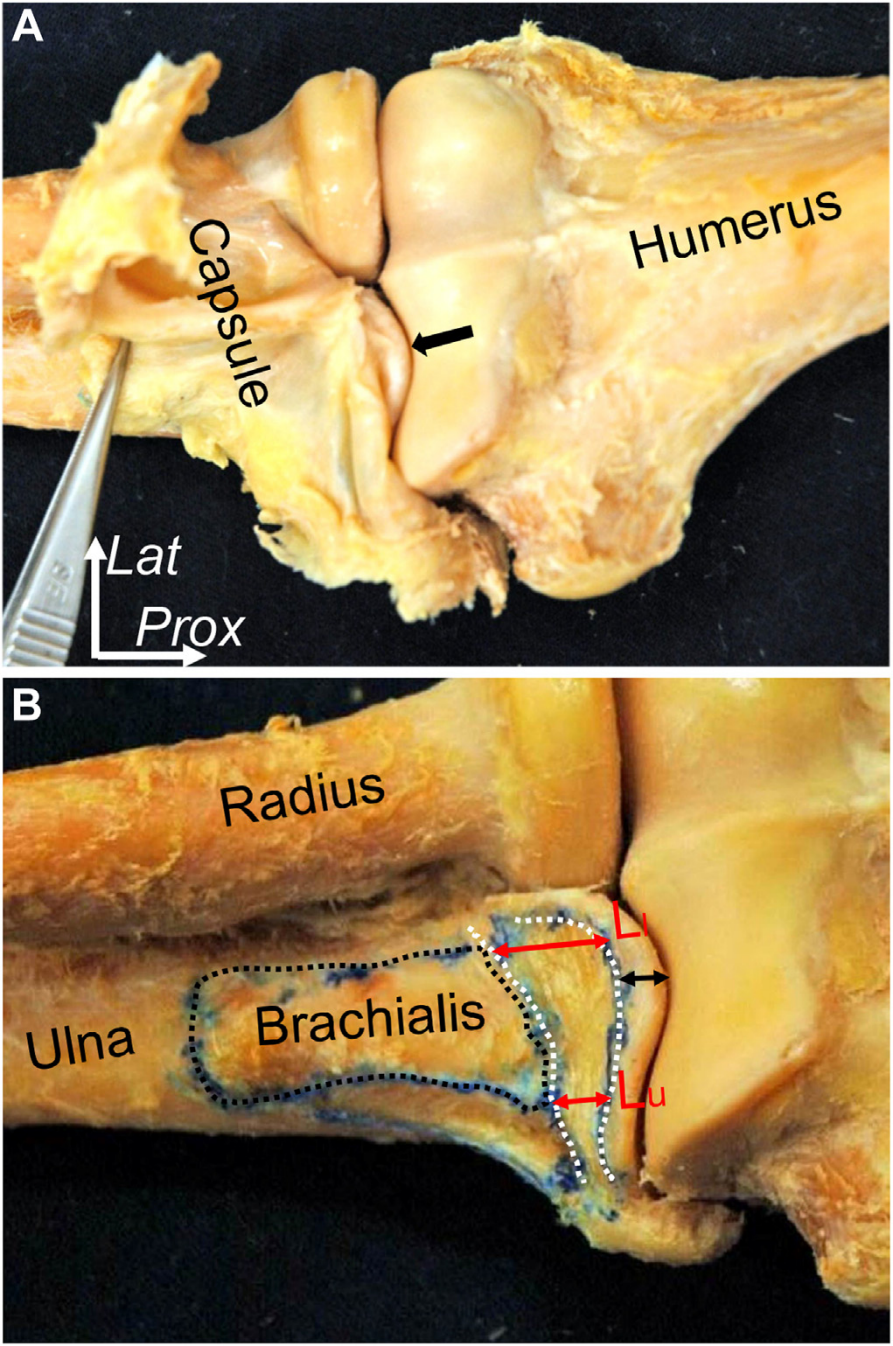
The attachment of the joint capsule to the coronoid process of the ulna. (**A**) The right elbow is shown after removing the surrounding muscles and detaching the joint capsule to be distally reflected by forceps. Black arrows indicate the tip of the coronoid process without the capsular attachment. (**B**) The joint capsule is removed, and capsular attachment (white dotted area) and insertion of brachialis muscle (black dotted area) are shown. Distance from the coronoid tip to proximal margin of capsular attachment is shown as the black double-headed arrow. The red double-headed arrows indicate the lengths of capsular attachment at the lateral (L_l_) and medial (L_m_) margin of the brachialis muscle insertion. *Prox*, proximal; *Lat*, lateral. (Reprinted from JSES International, 9/25, Haruhiko Shimura. Akimoto Nimura, Hisayo Nasu, Hitomi Fujishiro, Junya Imatani, Atsushi Okawa, Keiichi Akita, 1517-1522, 2016 with permission from American Shoulder and Elbow Surgeons).

**Figure 2:**
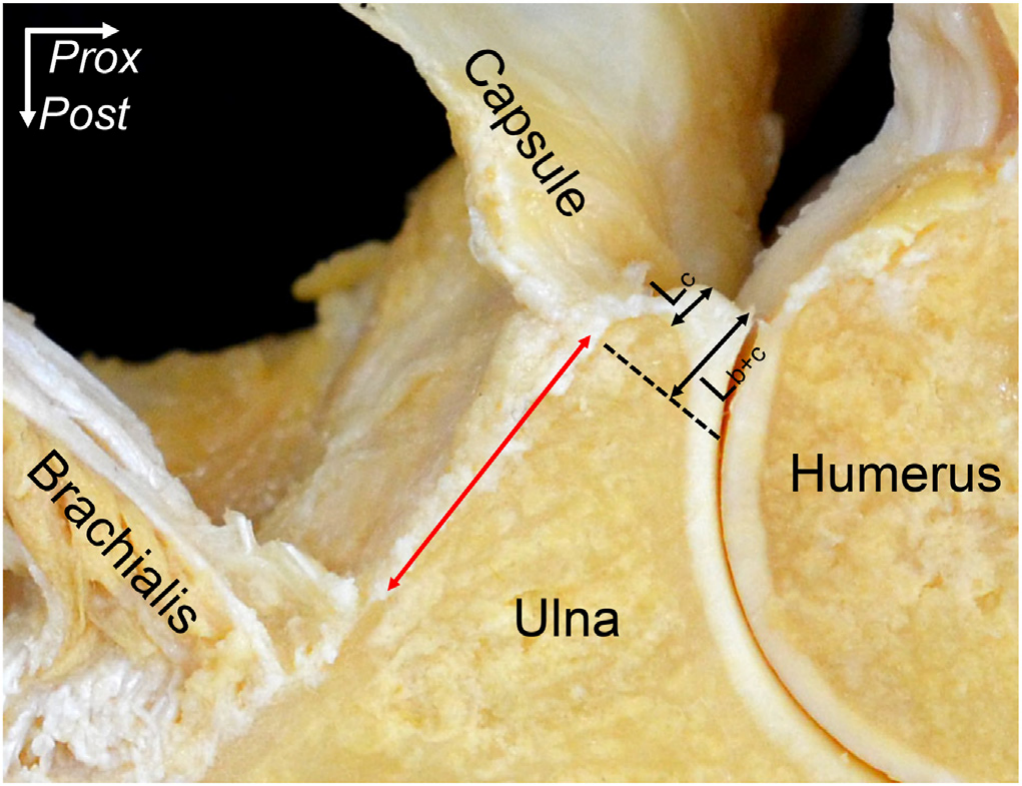
Length from the proximal margin of capsular attachment to the coronoid tip. The picture shows a sagittal section at the coronoid tip. The brachialis muscle and joint capsule are detached from the anterior aspect of the humerus and flipped to the distal. The distances between the tip of the coronoid process and the proximal edges of subchondral bone (L_c_) and the coronoid tip and proximal edge of capsular attachment (L_b + c_) are shown. The length of capsular attachment is shown as a red double-headed arrow. *Prox*, proximal. (Reprinted from JSES International, 9/25, Haruhiko Shimura. Akimoto Nimura, Hisayo Nasu, Hitomi Fujishiro, Junya Imatani, Atsushi Okawa, Keiichi Akita, 1517-1522, 2016 with permission from American Shoulder and Elbow Surgeons).

Regarding the tip of the coronoid process, the joint capsule does not attach at the proximal edge of the tip (Fig. 1, *A*).^27^ At the tip of the coronoid process, the distance from the proximal edge of the tip of coronoid process is roughly 6 mm proximal to the proximal border of the capsular attachment. At the medial and lateral borders of the brachialis muscle insertion, the lengths of capsular attachment are 6 mm and 12 mm, respectively (Fig. 1, *B*). When it comes to the type 1 fractures of the O’Driscoll classification,^22^ the cartilage thickness at the tip of the coronoid process is 2 mm (L_c_ in Fig. 2), and the distance from the coronoid tip to the proximal edge of the capsular attachment, including cartilage and subchondral bone is, 5 mm (L_b + c_ in Fig. 2). These findings suggest that subtype 1 of the tip fracture does not involve the capsular attachment, whereas subtype 2 does and is recommended for fixation.

Regarding the lateral side of the elbow joint, lateral epicondylitis, also known as tennis elbow, has been a mysterious pathology. The extensor carpi radialis brevis (ECRB) has been the most subject to the etiology of lateral epicondylitis.^20^ Additionally, the structures adjoining the joint capsule, such as the synovial fringe,^4^ and annular ligament,^2^^,^^3^ have been mentioned as potential causes of lateral epicondylitis. However, in terms of the pathogenic consideration of lateral epicondylitis, the joint capsule and its attachment have been neglected until recently. Recent anatomical papers in shoulder and knee joints described that relatively narrow attachments of joint capsules could have etiological relations with degenerative diseases such as rotator cuff tears and the extrusion of meniscus, respectively.^17^^,^^19^

**Figure 3:**
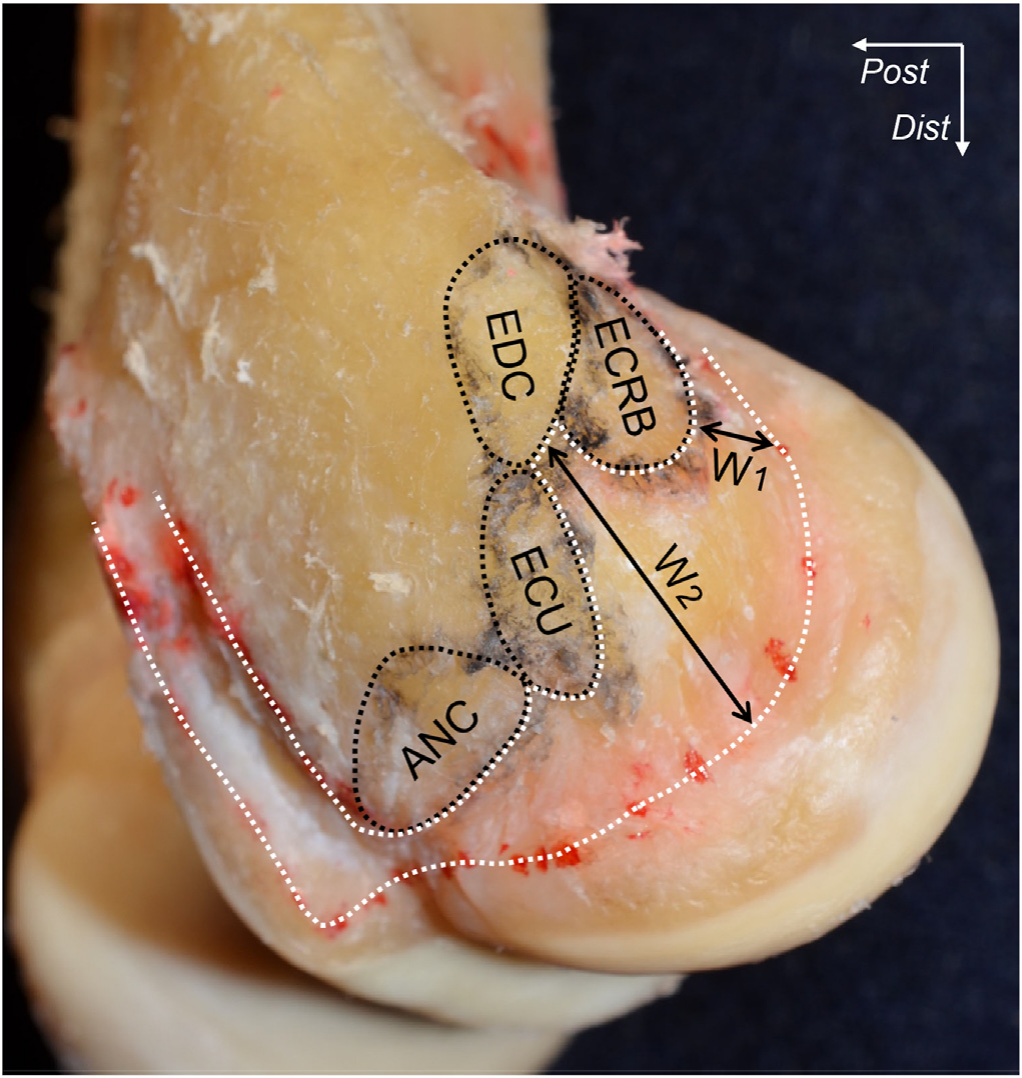
The regional variation of capsular attachment on the lateral aspect of the humerus. The origins of the superficial extensors are shown as black-dotted areas. The white-dotted area indicates the capsular attachment. Attachment widths at the anterior edge of the extensor carpi radialis brevis (ECRB) origin and distal edge of the extensor digitorum communis (EDC) origin are shown as W_1_ and W_2_, respectively. *ECU*, extensor carpi ulnaris; *ANC*, anconeus; *Dist,* distal; *Post,* posterior. (Fukai et al^7^).

In the first place, similar to the capsular attachment of the coronoid process of the ulna, the joint capsule attaches to the humerus with width, and these attachment widths differ according to their locations. On the lateral epicondyle of the humerus, the capsular attachment at the anterior part of the ECRB origin is narrower (W_1_ in Fig. 3) than one at the distal area of the ECRB and extensor digitorum communis (EDC) origins (W_2_ in Fig. 3). These findings could explain one reason for the initiation of lateral epicondylitis limited in ECRB origin.

## Consideration of lateral collateral ligaments of the elbow joint: what is a “ligament”?

Posterolateral rotatory instability of the elbow joint has been assumed as the main pathology of the complex elbow instability and is thought to be caused by a disruption of the lateral ulnar collateral ligament (LUCL). The LUCL is described as a posterior part of the lateral collateral ligament (LCL) of the elbow joint and a connection between the lateral epicondyle of the humerus and the supinator crest of the ulna. Strangely, regarding the LUCL, the existence and ulnar attachment have been inconsistently described in various papers.^8^^,^^12^^,^^23^

In general, the term “ligament” is assumed to be a band-like structure that connects bone to bone and is clearly separated from surrounding structures. In a histological textbook, a ligament is defined as being included in the dense connective tissues in which its fibers are more regularly oriented than aponeurosis and are less regularly arranged than tendon. However, the structural borders between tendons, ligaments, aponeuroses, and joint capsules are not clearly separated. Accordingly, recent studies in several joints have shown that ligaments could be interpreted as a part of surrounding structures such as tendons, aponeuroses, and joint capsules.^21^^,^^29^^,^^30^

**Figure 4:**
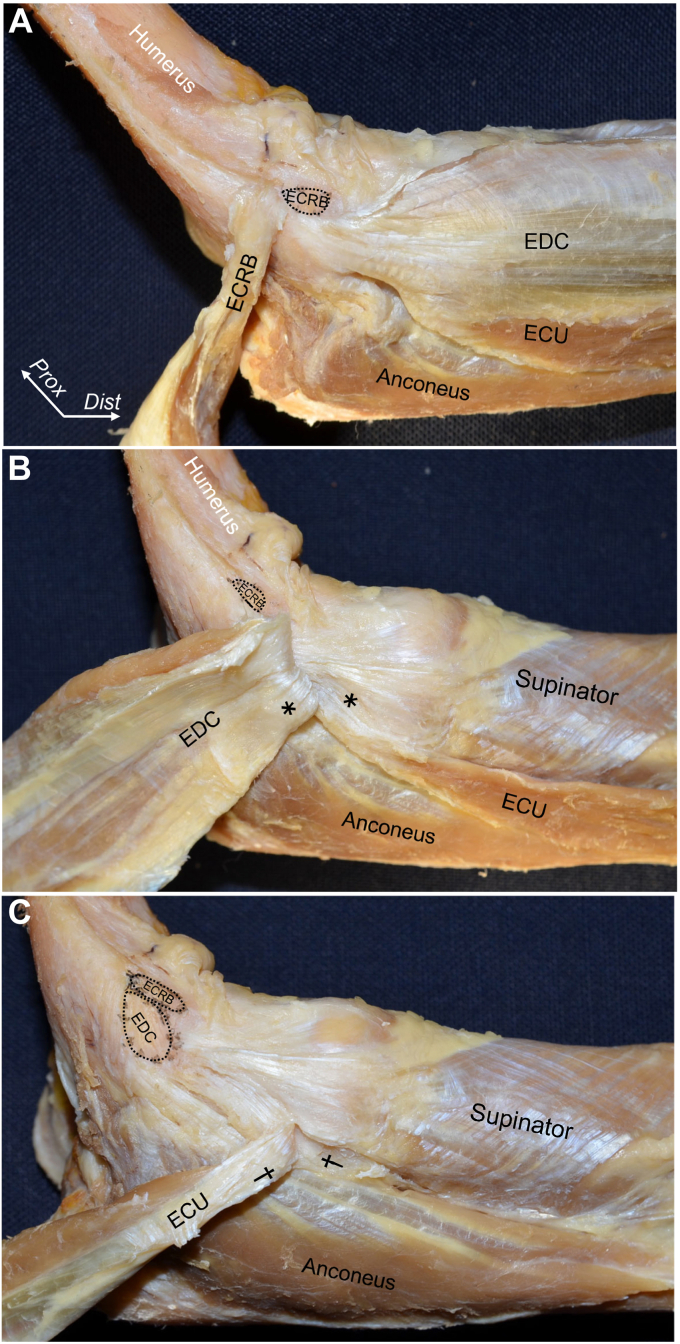
The connection between the superficial extensors and deeper structures. (**A**) The brachioradialis and extensor carpi radialis longus muscles are removed from the right elbow. The extensor carpi radialis brevis (ECRB) muscle is reflected to proximal. (**B**) ECRB is detached from the humeral origin, and the extensor digitorum communis (EDC) is reflected to proximal border. The *∗* indicates the deep aponeurosis of posterior EDC intermingles with the supinator origin. (**C**) EDC is detached from the humeral origin, and the extensor carpi ulnaris (ECU) muscle is reflected to proximal. The *†* indicates the deep aponeurosis of ECU is intermingles with the supinator origin. *Dist,* distal; *Prox,* proximal. (Fukai et al^7^).

**Figure 5:**
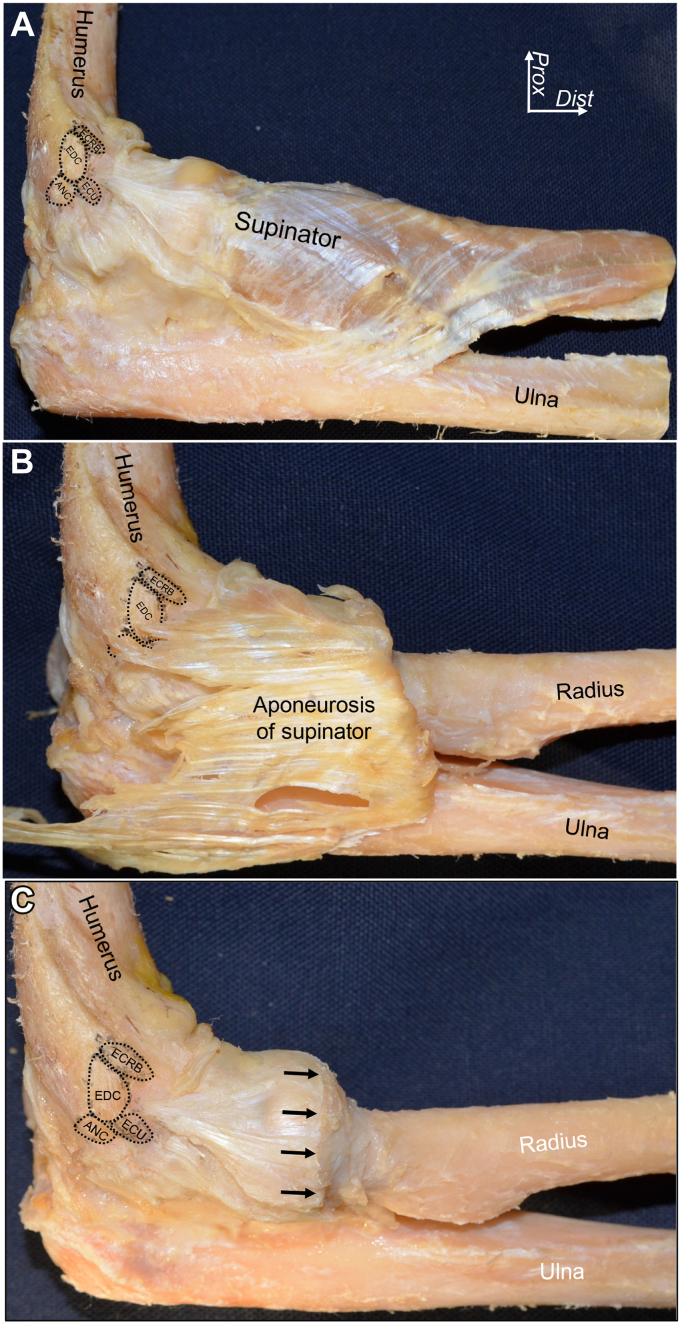
The capsuloaponeurotic membrane consists of the joint capsule and supinator aponeurosis. (**A**) The lateral aspect of right elbow after detaching the superficial extensor tendons. The black-dotted areas indicate the extensor origins on the humerus. (**B**) The muscular part of the supinator muscle is removed, and aponeurosis is reflected to the proximal border or margin. (**C**) The black arrows indicate the proximal border of the part where the supinator aponeurosis could be separated from the joint capsule. The supinator aponeurosis distal to black arrows are removed. *ECRB*, extensor carpi radialis brevis; *EDC*, extensor digitorum communis; *ECU*, extensor carpi ulnaris; *ANC*, anconeus, *Dist,* distal; *Prox,* proximal. (Fukai et al^7^).

**Figure 6:**
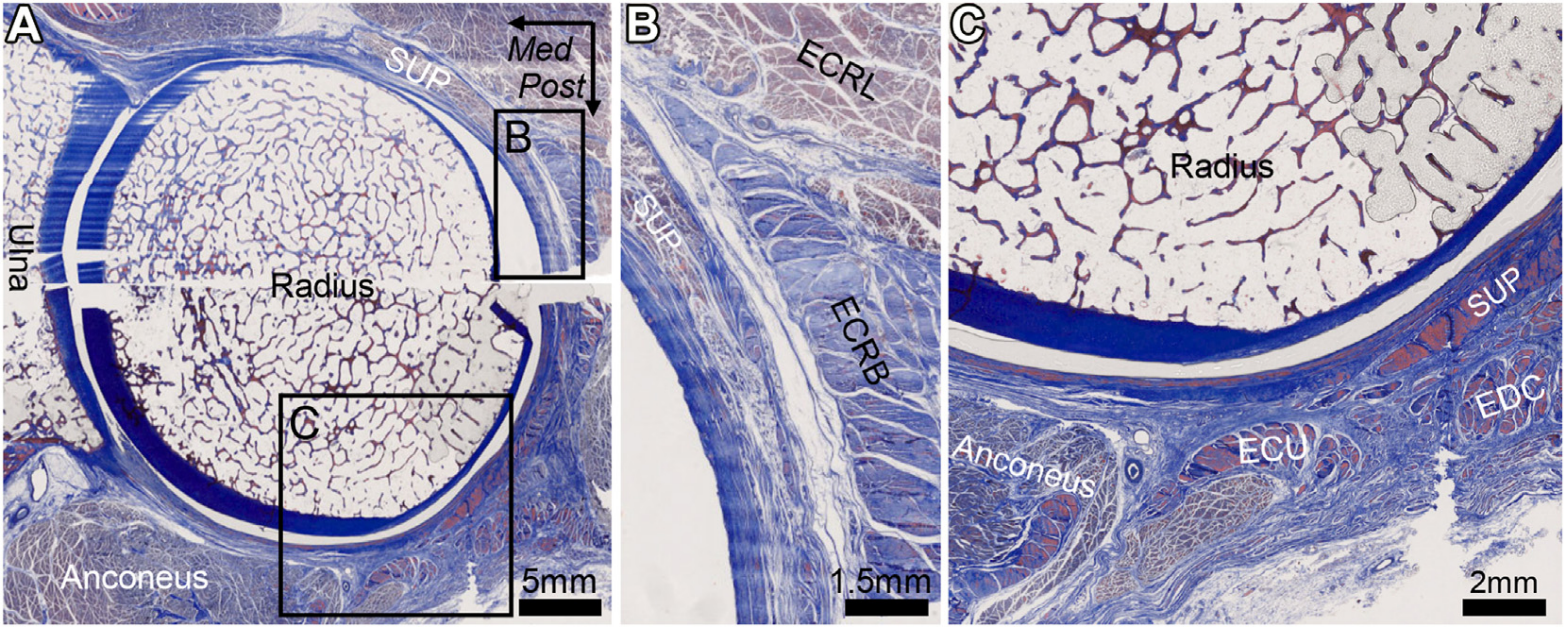
Connections between the superficial extensors, supinator muscle, and joint capsule. (**A**) The axial section at the radial head with Masson’s trichrome staining. (**B**) The black boxes in (**A**) are magnified as (**B** and **C**). *ECRL*, extensor carpi radialis longus; *ECRB*, extensor carpi radialis brevis; *EDC*, extensor digitorum communis; *ECU*, extensor carpi ulnaris; *SUP*, supinator; *Post*, posterior; *Med*, medial. Scale: A, 5 mm; B, 1.5 mm; C, 2 mm. (Fukai et al^7^).

Based on the anatomic concept described above, LCL and LUCL could be reconsidered by observation from the perspective of the relationship between the joint capsule and adjoining aponeuroses, which differs from specific ligaments. By detaching superficial extensor tendons, the deep aponeuroses of the ECRB and anterior part of the EDC are easily released from the joint capsule, including supinator aponeurosis (Fig. 4, *A*).^7^ In comparison with the ECRB and anterior part of EDC, the deep aponeuroses of the posterior part of EDC and the extensor carpi ulnaris (ECU) are mingled with the supinator aponeurosis (Fig. 4, *B* and *C*). By more deeply observing, the supinator aponeurosis originates from distal to the ECRB and EDC origin, expands to anterior and posterior, and intermingles with the joint capsule to develop a substantial complex, which is defined as the capsulo-aponeurotic membrane (Fig. 5, *A*). At the level of the radial neck, the superficial aponeurosis of the supinator was separated from the joint capsule by the interposing supinator muscle (Fig. 5, *B* and *C*). The axial section at the radial head level with Masson’s trichrome staining demonstrates that the posterior EDC and ECU are adjoining with the capsulo-aponeurotic membrane via dense connective tissues, while the ECRB tendon is distinct from the joint capsule by interposing loose connective tissues (Fig. 6).

**Figure 7:**
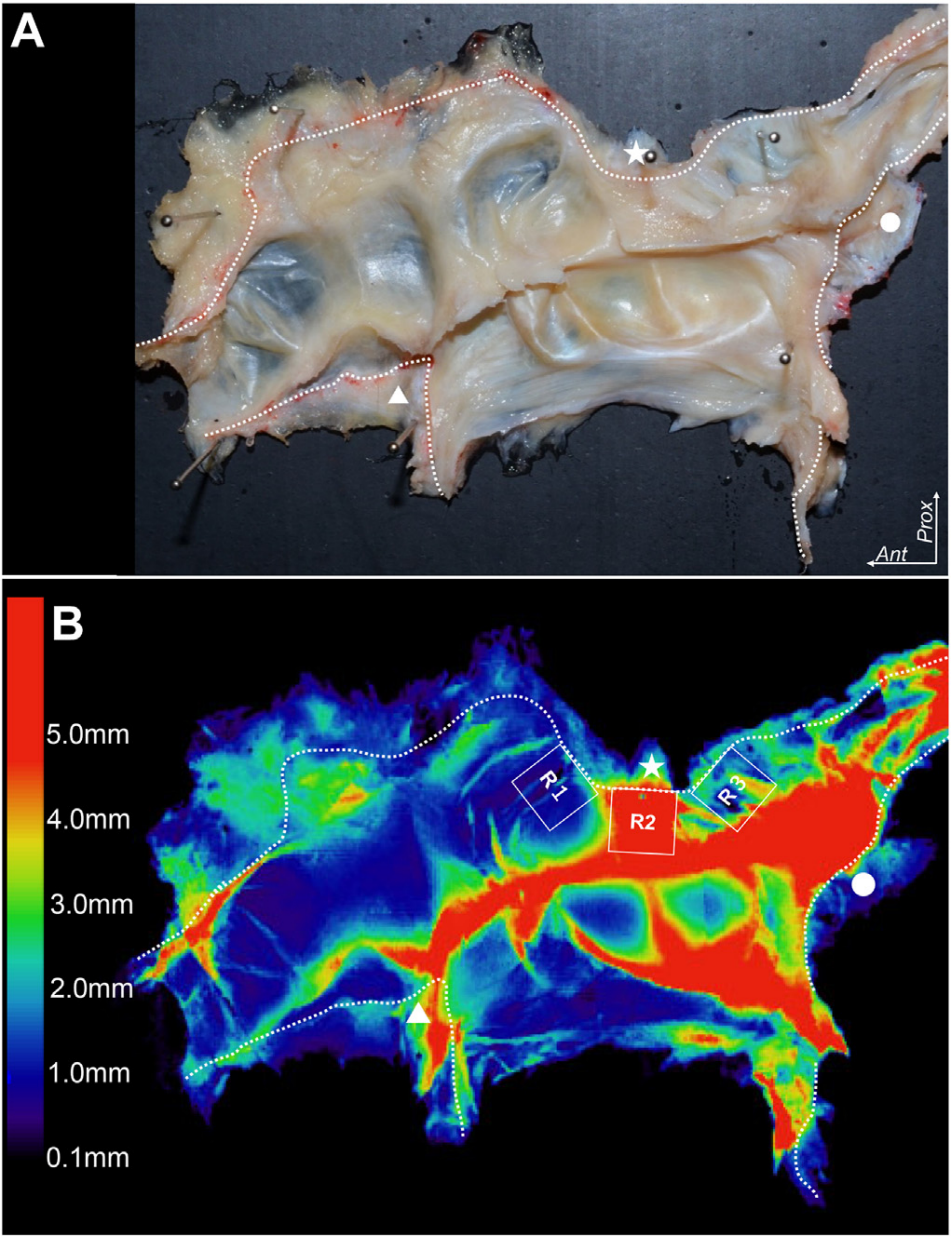
Distribution of capsular thickness at the lateral part of elbow joint. The joint capsule with supinator aponeurosis is flattened after detaching from bones (**A**) and shown as a heat map to visualize the thickness distribution after data processing (**B**). The white stars correspond to the lateral condyle of humerus. The white triangles correspond to the coronoid process of ulna. The white circles correspond to posterior part of the radial notch. (**A**) Inner aspect of the joint capsule. (**B**) The same specimen as A is shown. R1, R2, and R3 indicate the anterior, middle, and posterior parts of the radiocapitellar joint, respectively. The thicknesses are roughly demonstrated as the various colors in the left color bar. *Ant,* anterior; *Prox*, proximal. (Fukai et al^7^).

To visualize the thickness distribution in the joint capsule, the joint capsule was detached from the bones, planarly expanded, and imaged using micro-CT as the previously developed technique.^14^^,^^31^^,^^30^ The capsulo-aponeurotic membrane (R2 in Fig. 7, *B*) shows a brighter color than the anterior and posterior parts of the joint capsule (R1 and R3 in Fig. 7, *B*), ie, the capsulo-aponeurotic membrane is thicker than the anterior capsule underlying the trochlea and capitellum, and the posterior one underlying the anconeus muscle.

Based on the findings described above, the LCL, otherwise known as the radial collateral ligament, of the elbow joint could be interpreted as the capsulo-aponeurotic membrane, which consists of the joint capsules, including supinator aponeurosis. In addition, the deep parts of the EDC and ECU aponeuroses are connecting with the capsulo-aponeurotic membrane and making them seem cord-like structures. This band could be corresponding with the LUCL.

These perspectives have two clinical implications. First, we could propose a new reconstruction technique for the complex elbow instability on behalf of the LUCL reconstruction using band-like autologous tendons.^11^ Given that the posterior part of the capsulo-aponeurotic membrane mingles with the deep aponeuroses of EDC and ECU, we recommend three things to improve the clinical outcome of treatments for instability: 1) to use the triangular membranous graft rather than a band-like one; 2) to connect the graft with the lateral part of the coronoid process, distal to the lateral epicondyle of the humerus, and dorsal to the radial notch of the ulna; and 3) to stitch the remaining aponeuroses such as the EDC, ECU, and supinator in order to make them work as the “static-dynamic” stabilizer described below.^7^

Second, the pathology of lateral epicondylitis is relevant. Based on new perspectives regarding LCL, if the pathology initiated at the thin part of the joint capsule, which is located anterior to the ECRB origin, and posteriorly expanded to the capsulo-aponeurotic membrane, this structural deterioration could induce the subtle instability that denotes the poor clinical outcomes of treatment previously described.^5^^,^^18^

## Consideration of ulnar collateral ligaments of the elbow joint: static and dynamic stabilizers

Baseball pitchers are susceptible to ulnar collateral ligament (UCL) injuries to the elbow joint.^6^ The anterior bundle of UCL is believed to serve as the main or static stabilizer against valgus stress during throwing motions.^15^ The flexor-pronator muscles (FPMs), including the flexor carpi radialis (FCR), flexor carpi ulnaris (FCU), flexor digitorum superficialis (FDS), and pronator teres (PT), are also believed to contribute to dynamic stabilization. Even though the proximity of static and dynamic stabilizers in the medial part of elbow joint, the anatomical relationship has been rarely discussed.

**Figure 8:**
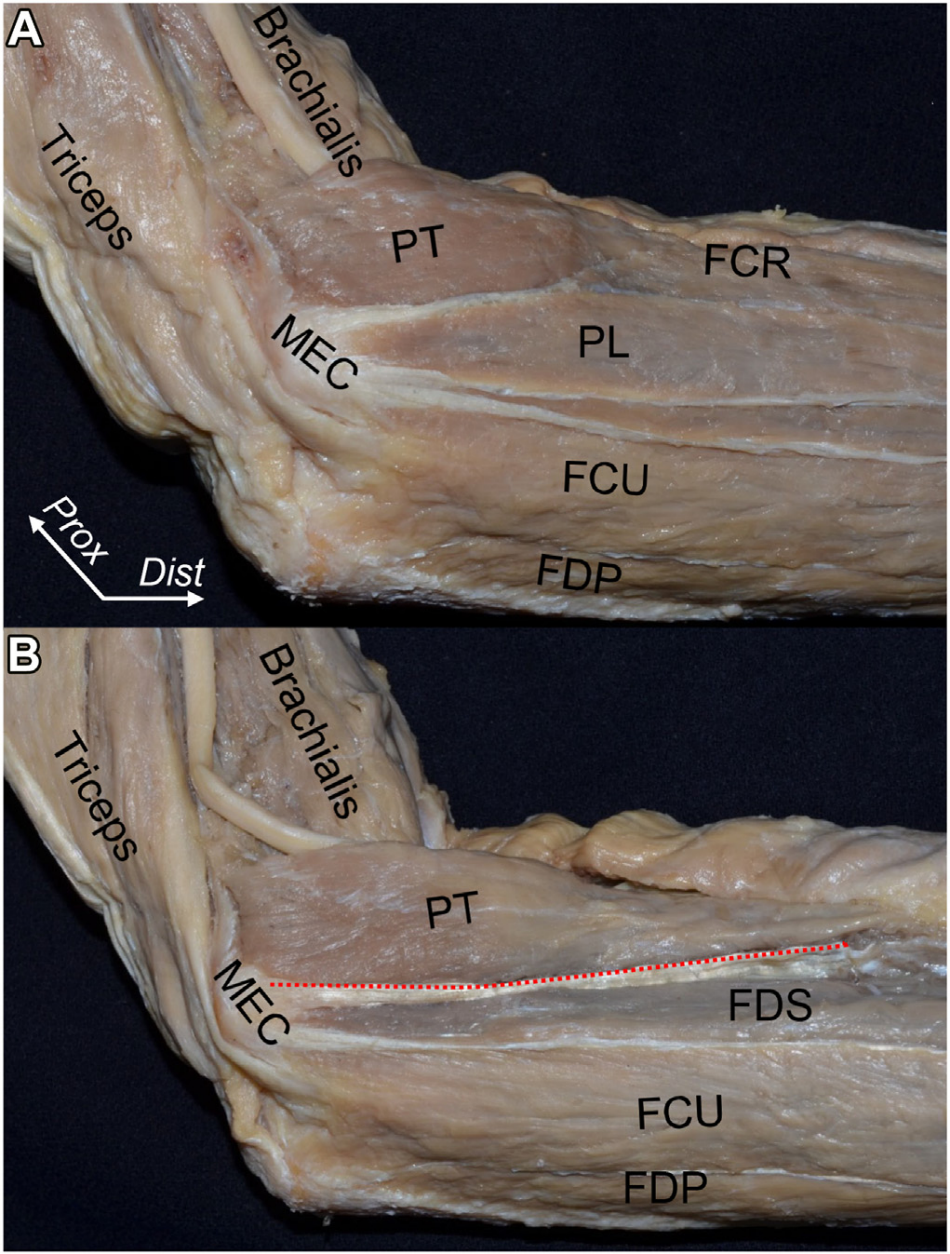
Superficial and deep layers of the flexor-pronator muscles (FPMs). The medial aspect of left elbow after removing skin and superficial fascia is shown. (**A**) FPMs have a common muscular origin at the medial epicondyle of the humerus (MEC). (**B**) The flexor carpi radialis (FCR) and palmaris longus (PL) muscles are detached. The tendinous septum between the pronator teres (PT) and flexor digitorum superficialis (FDS) muscles is shown as the red dotted line. *FCU*, flexor carpi ulnaris; *FDP*, flexor digitorum profundus; *Dist*, distal; *Prox*, proximal. (Modified from Hoshika et al^11^).

**Figure 9:**
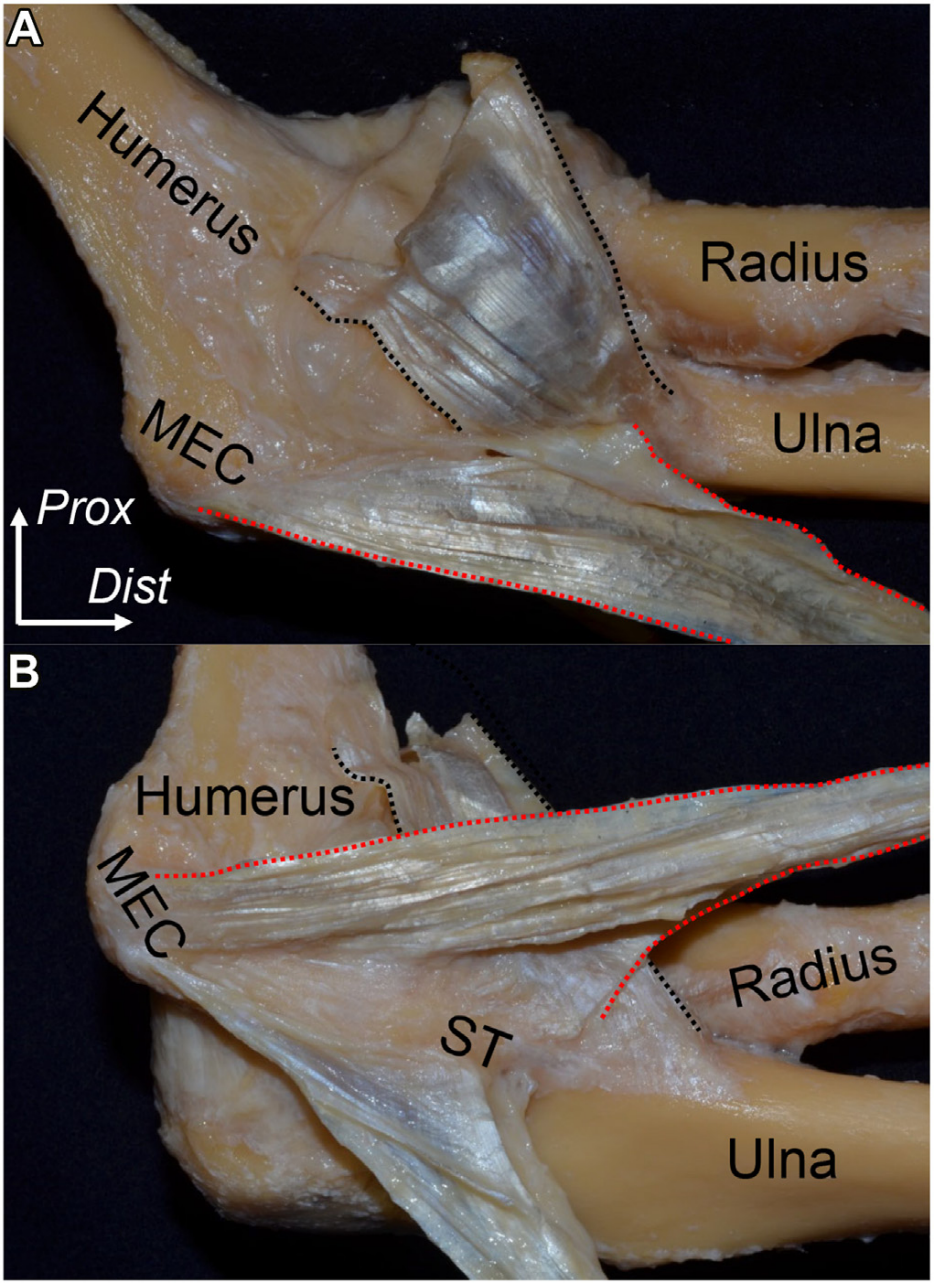
The tendinous septum connects with surrounding structures. From the left elbow in Figure 8, all muscular parts are removed. (**A**) The brachialis muscle aponeurosis (*black dotted area*) and the anterolateral aspect of the tendinous septum between the pronator teres and flexor digitorum superficialis muscles (*red dotted area*) are shown. (**B**) In the same elbow, the tendinous septum is reflected anteriorly. *MEC*, medial epicondyle of the humerus; *ST*, sublime tubercle; *Dist*, distal; *Prox*, proximal. (Modified from Hoshika et al^11^).

**Figure 10:**
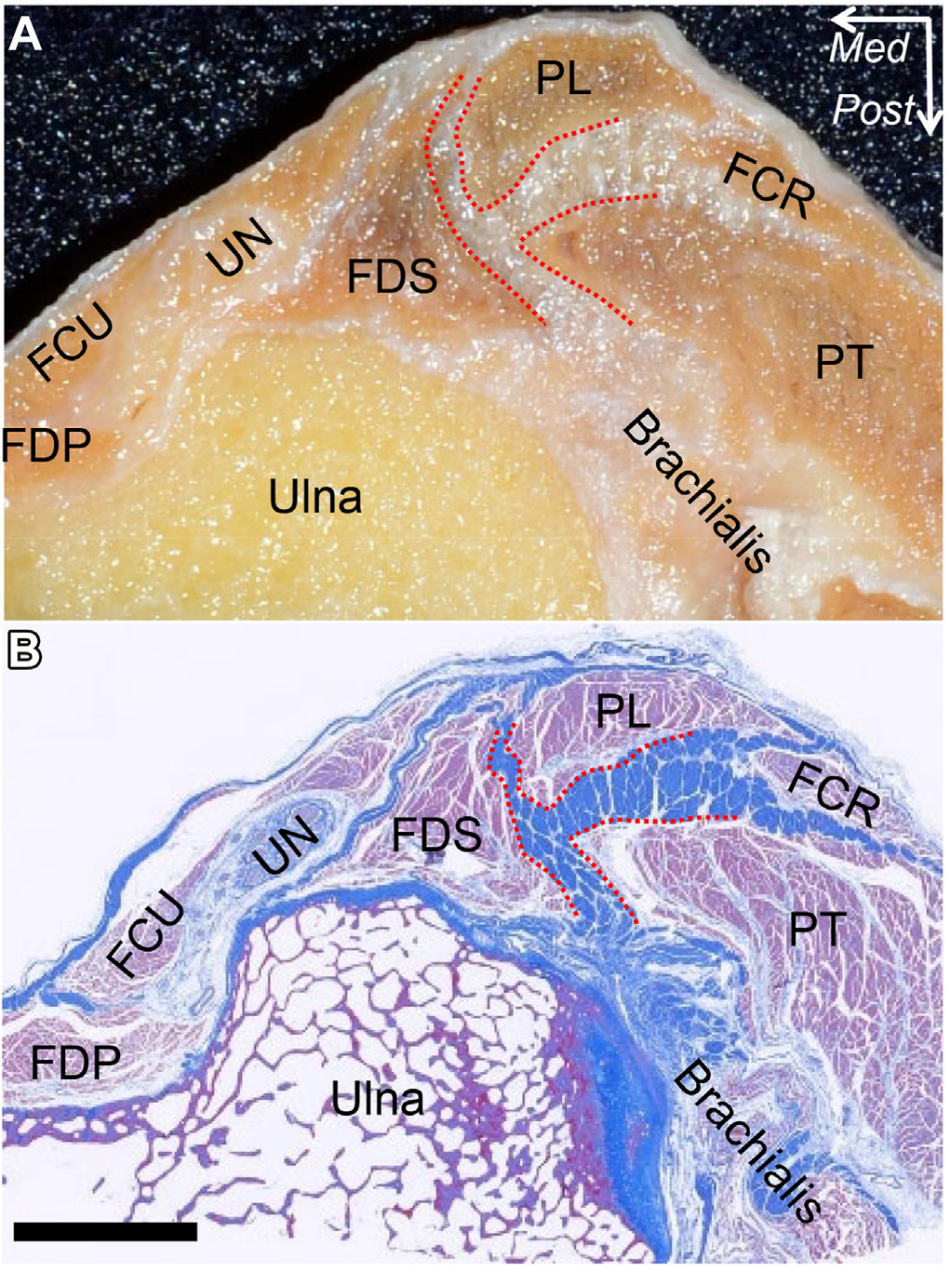
The axial sections at the sublime tubercle. (**A**) The macroscopic view of the axial section at t the sublime tubercle of ulna. The tendinous septum between the pronator teres (PT) and flexor digitorum superficialis (FDS) muscles is shown as the red dotted area. (**B**) Masson’s trichrome staining of the section of (**A**) is shown. *FCR*, flexor carpi radialis; *FCU*, flexor carpi ulnaris; *FDP*, flexor digitorum profundus; *PL*, palmaris longs; *UN*, Ulnar nerve; *Med*, medial; *Post*, posterior; Scale bar, 5 mm (Modified from Hoshika et al^11^).

At the medial epicondyle of the humerus, FPMs developed a common muscular origin. The palmaris longus and FCR overlie the PT and FDS muscles (Fig. 8, *A*).^10^ By removing the muscular portions, the connections between adjoining muscles are revealed. The FDS and PT muscles have a common tendinous septum (Fig. 8, *B*), and the brachialis tendon is partially connected with the base of the tendinous septum (Fig. 9, *A*). The tendinous septum between PT and FDS muscles originates from the anterior wall of the medial epicondyle, extends to the anterior base of the sublime tubercle of the ulna, and changes to the FDS deep aponeurosis, which overlies the joint capsule (Fig. 9, *B*). By histologically analyzing the axial section just distal to the tip of the coronoid process of the ulna, the tendinous septum between the FDS and PT muscles is joined to the tendinous portion of the brachialis muscle, transitioned to the FDS deep aponeurosis, and is all densely stained (Fig. 10, *A* and *B*)

Based on the anatomical knowledge described above, the medial part of the elbow joint is stabilized by the tendinous complex, which is composed of the tendinous septum between the FDS and PT muscles, the medial part of the brachialis muscle, and the FDS deep aponeurosis. In addition, the joint capsule underlies the deep surface of the tendinous complex. In other words, the anterior bundle of UCL could be understood as the grossly separated collagenous structure from the tendinous complex and joint capsule, if you similarly consider LCL and LUCL.

These anatomical viewpoints have the following implication: the stabilizing mechanism against valgus instability has been discussed as clearly separated between the static and dynamic stabilizers. Similarly, as with the LCL and LUCL described above, the aforementioned tendinous complex could work as a “static-dynamic” stabilizer. The FPMs have been thought to maintain the medial stability of the elbow joint.^16^^,^^24^^,^^28^ Among FPMs, PT, FDS, and FCU are assumed to stabilize the elbow joint against valgus force during pitching motion.^13^^,^^16^ By anatomical consideration of FPMs, the FDS function seems to have a particularly higher contribution to stability because the FDS origin adjoins both the tendinous septum and the connecting deep aponeurosis. Hoshika et al,^9^ showed that the individual finger flexion using isolated contraction of FDS muscle, specifically the second and third fingers, more effectively worked than other fingers in terms of narrowing the medial joint space, and claimed that injury care programs for overthrowing athletes should include FDS functional exercises.

## Conclusion

The current review focuses on several perspectives that previously had not been well understood. First, the width of the joint capsule bony attachments vary with location. Second, the structures, which have been generally called as “ligaments,” could be thought of as a part of periarticular aponeuroses and joint capsules. Therefore, ligaments have the potential to work as a “static-dynamic” stabilizer rather than a simple static one. Based on these anatomical concepts, we hope that some challenging pathologies could be cleared or innovative treatments could be developed in the future.

## Disclaimers:

Funding: This study was partly supported by a grant from 10.13039/100018590JA Kyosai Research Institute (Agricultural Cooperative Insurance Research Institute).

Conflicts of interest: The authors, their immediate families, and any research foundations with which they are affiliated have not received any financial payments or other benefits from any commercial entity related to the subject of this article.
